# Mechanism of furfural toxicity and metabolic strategies to engineer tolerance in microbial strains

**DOI:** 10.1186/s12934-023-02223-x

**Published:** 2023-10-28

**Authors:** S. Bilal Jilani, Daniel G. Olson

**Affiliations:** 1 Thayer School of Engineering, Dartmouth College, 15 Thayer Drive, Hanover, NH 03755 USA; 2 Center for Bioenergy Innovation, Oak Ridge National Laboratory, Oak Ridge, TN 37830 USA

**Keywords:** Hemicellulose, Acid pretreatment, Furfural, 5-hydroxymethyl furfural, Inhibitor, Hydrolysate, Xylose, Stress, Ethanol, Fermentation

## Abstract

Lignocellulosic biomass represents a carbon neutral cheap and versatile source of carbon which can be converted to biofuels. A pretreatment step is frequently used to make the lignocellulosic carbon bioavailable for microbial metabolism. Dilute acid pretreatment at high temperature and pressure is commonly utilized to efficiently solubilize the pentose fraction by hydrolyzing the hemicellulose fibers and the process results in formation of furans—furfural and 5-hydroxymethyl furfural—and other inhibitors which are detrimental to metabolism. The presence of inhibitors in the medium reduce productivity of microbial biocatalysts and result in increased production costs. Furfural is the key furan inhibitor which acts synergistically along with other inhibitors present in the hydrolysate. In this review, the mode of furfural toxicity on microbial metabolism and metabolic strategies to increase tolerance is discussed. Shared cellular targets between furfural and acetic acid are compared followed by discussing further strategies to engineer tolerance. Finally, the possibility to use furfural as a model inhibitor of dilute acid pretreated lignocellulosic hydrolysate is discussed. The furfural tolerant strains will harbor an efficient lignocellulosic carbon to pyruvate conversion mechanism in presence of stressors in the medium. The pyruvate can be channeled to any metabolite of interest by appropriate modulation of downstream pathway of interest. The aim of this review is to emphasize the use of hydrolysate as a carbon source for bioproduction of biofuels and other compounds of industrial importance.

## Background

Carbon neutral production of a molecule of industrial interest is desirable in order to minimize the release of additional carbon into the atmosphere incurred due to either combustion of the fossil fuels or in the mining and extraction of the compound of interest. Combustion of fossil-based fuels results in the greenhouse effect where the released carbon traps the heat radiated form the earth’s surface and causes an increase in the atmospheric temperatures and is detrimental to the functioning of the ecosystems. Among the gases released by the combustion process methane is the most potent green house gas and constitutes 20% of the gases generated by combustion of fossil fuels [1]. The production of ethanol over the past decade has increased at a rate of 3.8% [2] and in the year 2021, its consumption as a vehicular fuel in the US was 14,023 million gallons [3]. Thus, a bioderived molecule with usage as a transportation fuel will help to offset the carbon release in a significant manner. The process of refining sugars like glucose and xylose which are frequently used as carbon sources to produce industrial molecules is a cost intensive process and increases the production cost of the metabolite of interest [4]. For economically efficient production of a bioderived molecule of industrial utility, it is highly desired to utilize omnipresent plant biomass as a carbon source without significant refining of the trapped sugars [5]. However, before lignocellulosic biomass can be efficiently utilized as a carbon source for microbial metabolism it is necessary to make the carbon (both hexose and pentose sugars) bioavailable for microbial metabolism.

Plant cell wall is a complex structure consisting of cellulose, non-cellulosic polysaccharide matrixes like hemicelluloses and pectin, glycosylated proteins and lignin. The structural organization of higher plants consists of cellulose microfibrils cross linked by single-chain polysaccharides. The composition varies across different biomasses. On a dry weight basis, hardwood consists of mainly cellulose (50%), followed by hemicellulose (23%) and lignin (22%). Herbaceous plants and agricultural residues contain relatively higher amount of hemicellulose (30–33%) followed by cellulose (38–45%) and low level of lignin (10–17%) [6]. Lignocellulosic biomass contain three main classes of polysaccharides: cellulose, hemicellulose and pectins. The structural composition of each polysaccharide is as follows. Cellulose consists of unbranched D-glucose units bonded by β-1,4-glycosidic bonds while hemicelluloses are branched heteroploymers consisting of cellulans and polyuronides. Hemicelluloses form hydrogen bonds to the surface of cellulose fibrils. Cellulans are made up of hexosans (mannan, galactan and glucan) and pentosans (xylan and arabinan) [7–9]. Polyuronides, in addition, contain hexuronic acids and methoxyl, acetyl, and free carboxylic groups. Pectins are heteropolysaccharide defined by the presence of uronic acids as major components. Lignin is composed of polymer of phenylpropanoic acid in a complex 3-D structure and its monomers are bonded together by ether and C–C bonds [10]. Lignin has a random structure and is formed due to polymerization of free radicals. Both lignin and hemicellulose form a protective sheath around cellulose microfibrils which also contributes to the recalcitrance of the cellulose [11]. This review is focused on solubilization of the hemicellulosic fraction.

**Fig. 1:**
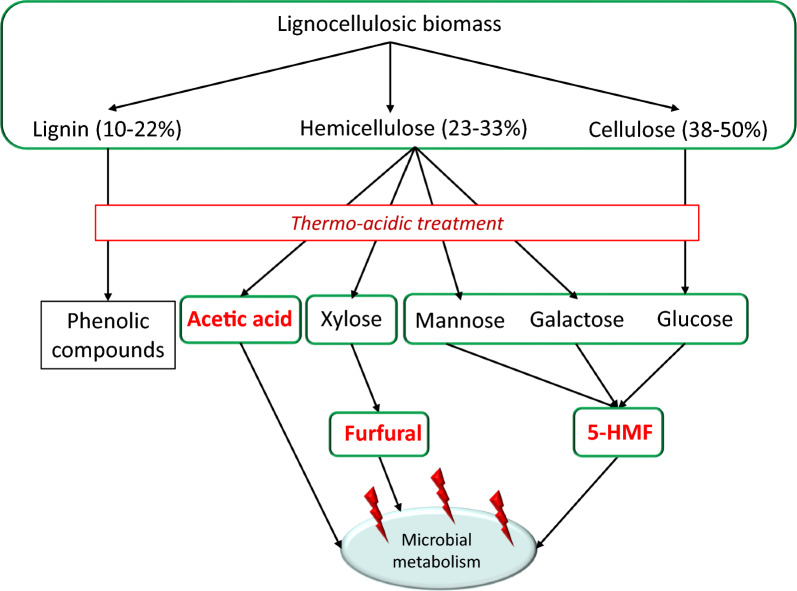
Composition of lignocellulosic biomass and products generated during deconstruction process. The proportion of sugars—xylose, mannose, galactose, glucose—and inhibitors—furfural, 5-HMF, acetic acid and phenolic compounds—varies according to the severity of thermo-acidic treatment. Relatively stringent conditions result in higher yield of both sugars and inhibitors

One of the steps in making lignocellulosic carbon efficiently bioavailable is to process it with dilute acid under high temperature and pressure and is commonly referred to as thermo-acidic treatment (Fig. 1). The liquid thus obtained contains the hydrolyzed sugars and is referred to as hydrolysate. It results in release of the recalcitrant hemicellulose portion of lignocellulose and solubilizes the pentose fraction. Pentose sugars can constitute 5–25% dry weight of lignocellulosic biomass [12–16] with xylose being the second most abundant sugar after glucose. Solubilization of sugars is directly proportional to the severity of thermo-acidic treatment which also leads to a proportional increase in concentration of inhibitors formed due to breakdown of the sugars [16–21] under harsh conditions. These inhibitors are detrimental to microbial metabolism [22–25] and to overcome inhibitor challenge the cell diverts its metabolic machinery towards detoxification which in turn result in significant loss of productivity as synthesis of the desired industrial product can only take place once inhibitors have been removed from media. It increases production cost of the compound and makes the process economically non-feasible especially in case of low value compounds such as ethanol.

For cost efficient conversion of solubilized sugars present in the hydrolysate, it is desirable to have microbial strains which can maintain optimum metabolic activity in presence of the inhibitors. In terms of volume, the major inhibitory compounds are furfural, 5-hydroxymethyl furfural (5-HMF) and acetic acid [26, 27]. Furfural and 5-HMF are generated due to dehydration of pentose and hexose sugars, respectively [28, 29]. Concentration of furfural in hydrolysate varies depending upon the source of biomass and severity of the treatment [17]. In major crop residues it has been reported to be: wheat straw (5–7.1 mM), barley straw (30 mM), sugarcane bagasse (20 mM) and corn stover (115 mM) [30]. Though 5-HMF is also detrimental to microbial metabolism it is relatively less toxic as compared to furfural. Acetic acid is formed due to hydrolysis of acetyl groups which are attached to the hemicellulose and lignin components of the biomass [27]. Due to the presence of multiple inhibitors in the hydrolysate a microbial strain encounters multiple stressors due to which the metabolism is overwhelmed and leads to either a microbicidal or a growth static effect on the cells. Noteworthy studies involving genomics and adaptive laboratory evolution [31–34] based approaches have been successfully used to reverse engineer the tolerance traits in different microbial strains and are not the focus of present review. Aromatic inhibitors derived from lignin degradation are also not in the scope of this review and interesting studies on modes of toxicity and tolerance against the same are described elsewhere [22, 35, 36]. The present review is centered on the major furan inhibitors—furfural and 5-HMF—and their modes of toxicity. As described further, furfural is relatively more toxic to microbial metabolism and is thus considered as the representative furan inhibitor. Mode of furfural toxicity and tolerance studies in microbial strains form the crux of this review. The similarity between furfural and acetic acid toxicity and tolerance is discussed followed by furfural tolerance studies reported in different microbial strains. It is important to note that though the research describes tolerance with response variable being a measure of increased ethanol titers, the robust microbial strains harbor an efficient lignocellulosic carbon to pyruvate conversion machinery. The pyruvate has the potential to be diverted to any desired metabolite of interest upon appropriate genetic manipulation.

### Furan inhibitors

**Fig. 2:**
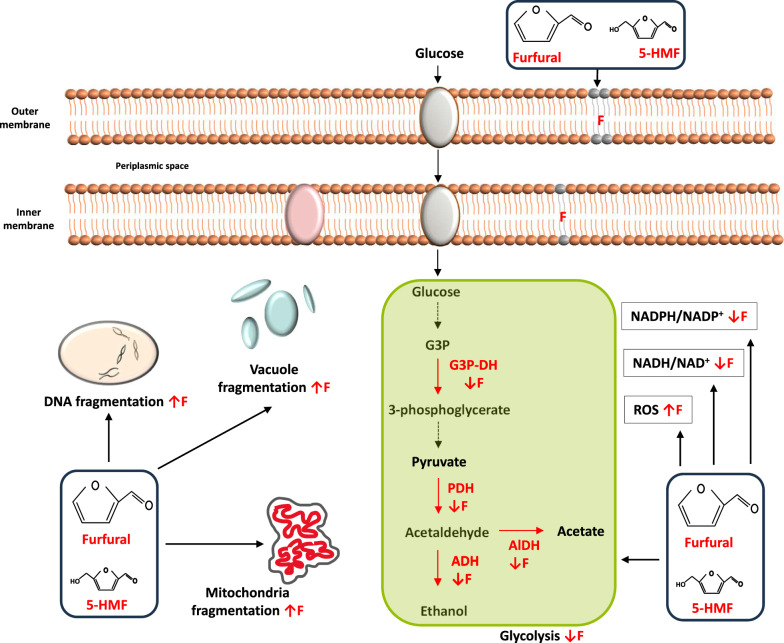
Cellular targets of furfural and 5-HMF. Generalized diagram of microbial metabolism with furfural targets highlighted in red. ↓ represents downregulation of function while ↑ represents an increase. Furfural is the major inhibitor as compared to 5-HMF. *F* represents furfural, *ROS* reactive oxygen species, *G3P* glyceraldeyde-3-phosphate, *G3P-DH* glyceraldehyde-3-phosphate dehydrogenase, *PDH* pyruvate dehydrogenase, *AlDH* aldehyde dehydrogenase, *ADH* alcohol dehydrogenase

Both furfural and 5-HMF are aldehydes whose toxic effects on microbial cells have been reported. Both inhibitors diffuse through the microbial membrane and have detrimental effects on its functioning. It leads to an increase in the reactive oxidative species in the cytoplasmic milieu, fragmentation of DNA, mitochondria and vacuole, redox imbalance and inhibition of enzymes of the glycolytic pathway (Fig. 2). It has been observed that acetaldehyde at a concentration of 1.56 mM can induce single strand breaks while at a concentration of 100 mM can induce double stranded breaks in DNA. Importantly the DNA damage induced by acetaldehyde is irreversible and no repair was observed even after 120 min on human lymphocytes [37]. Members of *Enterobacteriaceae* like *Escherichia coli* harbor genes whose products can be beneficial in neutralizing the harmful effect of acetaldehyde. A gene from acetaldehyde resistant *E. coli* strain VU3695 has been defined which encodes for glutathione and NAD dependent formaldehyde dehydrogenase. The gene showed homology with four alcohol dehydrogenases isolated from rat liver (63.2%), humans (62.7%) and horses (62.4%) and the catalytic action of its translated product results in degradation of formaldehyde into inactive S-formylglutathione in presence of glutathione and NAD [38].

Furfural is an industrial solvent and its production and physical properties defined as early as in 1948 [28]. The yield of furfural has been reported to be directly proportional to the xylan content of biomass, acidity and an increase in temperature with rice hull having potential to convert 12–13% of pentose content to furfural. Furfural has an almond like odor with oily texture [28]. However, from a microbial perspective it is considered a metabolic inhibitor and its hexose counterpart is 5-HMF [39]. Among the mix of inhibitors generated by woody biomass the concentration of furfural and 5-HMF correlated well with the fermentability of the hydrolysate [40]. This suggests that furan inhibitors form the key components of toxins generated due to pretreatment of biomass. Classical studies were performed wherein the synergistic effect of the inhibitors derived from dilute acidic treatment of lignocellulosic biomass was observed on *Saccharomyces cerevisiae* [41, 42] and *Escherichia coli* [39, 43, 44] and also reported that toxicity of other inhibitors on microbial growth is exacerbated in presence of furfural. The *E. coli* IC_50_ value of furfural and 5-HMF is reported as 2.9 and 3.8 g/L, respectively, which indicates that furfural is more toxic [39]. As part of microbial detoxification mechanism, both furans are converted to their respective alcohols which are relatively less toxic to microbial metabolism. Furfuryl alcohol with a IC_50_ = 4.0 g/L is relatively less toxic as compared to its aldehyde parent furfural [44]. As explained in following sections, furfural exerts toxic effect on multiple cellular metabolic targets. A detailed description of furfural metabolism follows.

### Influence of furfural on the glycolytic pathway

Furfural has been reported to inhibit glycolytic pathway enzymes—glyceraldehyde-3-phosphate dehydrogenase (GAPDH) and alcohol dehydrogenase (ADH) and to a lesser extent hexokinase in *Saccharomyces cerevisiae*. As compared to the control, percent specific activity in presence of 1 g/L furfural for hexokinase, triose phosphate DH and alcohol DH was 89, 23 and 52%, respectively [45]. Another study reported a far potent inhibitory effect of furfural on glycolytic enzymes. In *Saccharomyces cerevisiae*, furfural at a concentration of 0.12 g/L reduced activity of both aldehyde dehydrogenase (AlDH) and pyruvate dehydrogenase (PDH) by 80% and at 1.0 g/L led to the reduction in activity by more than 90%. On the other hand, ADH appeared to be resistant to the inhibitor which maintained 90% of its activity at 0.12 g/L and at 40% in presence of 4 g/L. While activities of AlDH and PDH at 4 g/L was only 5 and 0.2%, respectively. The estimated *V*_*max*_ and *K*_*m*_ values of ADH for furfural were reported to be 0.0442 µmol/min and 1.83 mM, respectively, and follows Michaelis Menton kinetics with non-competitive inhibition. The estimated *V*_*max*_ and *K*_*m*_ values of AlDH towards furfural was reported to be 0.0010 µmol/min and 4.89 µM, respectively, and follows competitive model of inhibition. While the *V*_*max*_ and *K*_*m*_ values of PDH against furfural was 0.0089 µmol/min and 18.7 µM, respectively, and followed non-competitive model of inhibition [46] which suggests that PDH is comparatively a less preferred target enzyme for furfural inhibition. The major difference among PDH, ADH and AlDH is both ADH and AlDH can directly reduce furfural while PDH cannot. The reason might be that the substrate for PDH is a simple alpha keto acid (pyruvate) and due to stearic constraints, the active site of the enzyme is not able to accommodate the furan ring of furfural. While both ADH and AlDH use acetaldehyde as a natural substrate and can utilize furfural as a substrate since it is an aldehyde and the furan ring does not face stearic constraints in the active site of the respective enzyme. The significant difference in the enzyme sensitivity between the two studies can be explained as the earlier one [45] used a cell free extract to assess indirect measurement of respective enzyme activity without a clearly defined methodology. On the other hand, the latter study [46] used the classical methodology of purified enzymes and accessed activity by monitoring the 340 nm signal corresponding to NADH concentration.

In order to further investigate the role of glycolytic pathway genes in *S cerevisiae*, a genomic study-based approach was pursued with screening of a mutant library of *S. cerevisiae* for ability of the mutants to tolerate furfural. It was found that mutants deficient in pentose phosphate metabolism—*ZWF1*, *GND1* and *RPE1*—had reduced ability to metabolize furfural. At the end of 96 h, the media containing respective mutants had 45, 52 and 13% of the original furfural still present in the media [47] while no furfural could be detected in the wild type parent. Furfural also results in redistribution of carbon flux in the EMP pathway. Formation of glycerol is one strategy which microbial cellular machinery employs to regenerate NAD^+^. It has been reported that presence of furfural causes a decrease in glycerol titers under anaerobic conditions [41] with concomitant increase in ethanol titers. The higher ethanol titers were suggested to be due to the growth arrest caused by the presence of furfural in the medium where the cellular machinery instead of diverting glucose carbon for increase in biomass channels it towards formation of ethanol. On the other hand, it has also been reported that the same inhibitor also results in a decrease of ethanol formation. In one study where 4 g/L furfural was added to exponentially growing batch culture of *S. cerevisiae* a reduction in ethanol formation was observed. The rate of specific ethanol production fell from 1.6 (± 0.1) to 0.5 (± 0.2) g/g/h. The specific growth rate ($\mu )$ also decreased from 0.4 to 0.03 (± 0.02) /h. And even after complete removal of furfural from media, µ increased to 0.13 (± 0.03) /h only [48] which represents 67.5% repression of growth rate and suggests that furfural can cause long term repression of microbial metabolism even after removal of the stress.

### Influence of furfural on DNA

DNA damage by furfural is also extensively reported in scientific studies. It has been reported that a proportionate decrease in transformation efficiency of plasmid occurs as a result of increase in furfural concentration [49] in the medium. A study was performed in order to elucidate the mechanism behind the loss of transformation efficiency. Alkaline unwinding assay is a technique used to quantitate the amount of strand breakage in cellular DNA. The assay is based on the principle that upon exposure to the alkaline conditions (pH > 11.5) the double helix DNA molecule undergo strand separation at each strand break [50]. Using this technique, it was reported that (i) the amount of duplex DNA remaining in the sample decreased with an increase in furfural concentration (ii) with increase in time the furfural treated samples showed a consistent increase in the number of breaks formed per unit of DNA (iii) furfural treated DNA samples were hydrolyzed by the single strand specific S_1_ nuclease [51]. Using restriction enzyme digestion, the same study also provided evidence that furfural specifically targets the AT rich regions of DNA. These findings are supported by a later independent study where increasing the *denovo* biosynthesis of DNA via plasmid based overexpression of *thyA* results in increased tolerance against furfural [52]. *thyA* gene encodes for thymidylate synthase which converts dUMP to dTMP in a multistep process involving reductive methylation of 2’-deoxyuridine-5’-monophosphate (dUMP) using the co-substrate N5,N10-methylenetetrahydrofolate as a donor and producing dTMP and 7,8-dihydrofolate. Alongwith other genes, *thyA* was also reported in a genome wide mapping study to identify genes conferring tolerance against furfural [34]. These studies together suggest that furfural induced DNA damage can be compensated by increased de novo synthesis of pyrimidines.

### Influence of furfural on redox biology

Furfural also leads to redox stress in microbial cell and reversing the effect of oxidative stress is a NADPH intensive process. In case of *S. cerevisiae* it was reported that after 8 h in defined media, furfural at concentrations of 25 and 50 mM leads to formation of reactive oxygen species (ROS) in 31 and 36% of the cells, respectively [53]. In the absence of furfural only 10% of the cells displayed ROS stress. At 25 mM concentration, the vacuoles appeared to be lobular and fragmented into two to four medium sized vacuoles. The same study also reported furfural induced damage to mitochondria. In presence of 25 mM furfural, the mitochondria appeared fragmented (41%) and aggregated (9%) to one side of the cell while in the control cell, in absence of furfural, displayed normal tubular morphology (80%). In another study it was reported that 45 mM 5-HMF also leads to accumulation of ROS species in the yeast cells [54]. In complex media with 20 g/L glucose and 50 g/L xylose, under anaerobic cultivation conditions and sublethal concentration of furfural (4.2 mM) and 5-HMF (10.3 mM), the cofactor balance of *S. cerevisiae* strain VTT-10883 was determined [55]. The presence of inhibitors decreased the steady state level of NADH from 0.48 (± 0.08) to 0.20 (± 0.06) µmol/g DW and the concentration of NADPH decreased from 0.55 (± 0.17) to 0.14 (± 0.01) µmol/g DW. However, it was reported that cellular ATP pools are relatively resilient to the furan inhibitors. There was marginal effect on intracellular ATP concentration which in presence of inhibitors was 5.96 (± 0.41) as compared to 7.36 (± 0.36) in absence of the inhibitors. A detailed analysis of the intracellular cellular redox cofactors is presented in Table 1. Similarly a complete analysis of the intracellular adenylate pool, energy charge and ATP yield is presented in Table 2.

**Table 1:** Intracellular concentrations of redox co-factors at steady-state from chemostat cultivations with and without HMF and furfural added to the feed-medium

	[NAD^+^]	[NADH]	Catabolic reduction charge	[NADP^+^]	[NADPH]	Anabolic reduction charge
Control	3.07 ± 0.63	0.48 ± 0.08	0.13 ± 0.00	0.61 ± 0.07	0.55 ± 0.17	0.46 ± 0.11
Inhibitors	2.26 ± 0.70	0.20 ± 0.06	0.08 ± 0.00	0.37 ± 0.09	0.14 ± 0.01	0.27 ± 0.02

Concentrations are in µmol (g DW)^−1^ (reproduced from [55])

**Table 2:** Intracellular concentrations of ATP, ADP, AMP, energy charge (E_c_) and ATP yield (Y_ATP_) at steady-state from chemostat cultivations with and without HMF and furfural added to the feed-medium

	[ATP]	[ADP]	[AMP]	E_c_	Y_ATP_ g DW (mmol ATP)^−1^
Control	7.36 ± 0.36	1.87 ± 0.13	0.25 ± 0.01	0.87 ± 0.00	11.0 ± 0.48
Inhibitors	5.96 ± 0.41	1.84 ± 0.11	0.30 ± 0.03	0.85 ± 0.00	12.4 ± 0.14

Concentrations are in µmol (g DW)^−1^ unless stated otherwise (reproduced from [55])

The importance of cellular redox in conferring tolerance against both furfural and 5-HMF is further demonstrated by analysis of gene expression of a furan tolerant *S. cerevisiae* strain NRRL Y50049 [56]. The results pointed to enhanced transcript levels of *ZWF1* and consequently of *GND1*, *GND2* and *TDH1* which are involved in generation of NAD(P)H. These genes are key players of the PPP which is responsible to significantly fulfill the requirements of cellular NADPH pools.

### Metabolic engineering strategies to confer tolerance against furfural

With the intention of making robust microbial strains to improve ethanol productivity, quite a few studies have been conducted to identify the mechanisms of resistance against furfural and 5-HMF as summarized in Table 3. The studies described in this review used ethanol as a response variable to measure tolerance against the furan inhibitors. It is important to emphasize that the respective robust strain harbor an efficient glycolytic machinery which can convert lignocellulosic carbon (under stressful conditions) into pyruvate which in turn can be channeled towards biosynthesis of any metabolite of interest upon appropriate genetic intervention.

**Table 3:** Genes involved in metabolism of furfural in microbial biocatalysts

Organism	Conc	Target gene	Function	Culture conditions	Parameter tested^∏^	Reference
*E. coli*	1.5 g/L	*thyA↑, lpcA↑, groESL↑*	*thyA* – thymidylate synthase; *lpcA* – D-sedoheptulose 7-phosphate isomerase; *groESL* – chaperonin complex induced under heat shock	MOPS minimum medium with glucose	Biomass increase^#^: lpcA ~ 400% groESL ~ 100%	[34]
	10 mM	*thyA↑*	-As before-	AM1 minimum medium with xylose	Ethanol titer: ND*; ~ 30 g/L	[52]
	1.5 g/L	*thyA↑, ahpC↑, yhjH↑*	*ahpC* – component of alkyl hydroperoxide reductase; *yhjH* – c-di-GMP phosphodiesterase, regulation in switch from flagellar motility to sessile	MOPS minimum medium with glucose	Biomass increase^#^: *yhjH* ~ 300%, *ahpC* ~ 80%	[33]
	1.25 g/L	*mdtJI↑, sugE↑*	*mdtJI* – components of multidrug efflux transporter; *sugE* – proton coupled guanidium transporter	AM1 minimum medium with xylose	Biomass: ND*; *mdtJI*, *sugE* OD_600_ ~ 1.5 Ethanol productivity: 0.19 g/L/h*; *mdtJI* 0.42 g/L/h	[65]
	10 mM	*puuP↑, potE↑*	Proton dependent putrescine transporters	AM1 minimum medium with xylose	Ethanol titer: ND*; *puuP*, *potE* ~ 42 g/L	[66]
	1 g/L^+^	*∆pgi*	Interconversion of G6P to F6P in glycolysis	AM1 minimum medium with glucose-xylose mixture	Ethanol titer: ~ 2 g/L*; *pgi* ~ 20 g/L	[68]
	1 g/L	*∆yqhD, ∆dkgA*	*yqhD* – aldehyde reductase; *dkgA* – methylglyoxal and beta-keto ester reductase	Acid treated sugarcane bagasse	Double mutant with 3 and tenfold higher biomass and ethanol, respectively, as compared to the control	[69]
	1 g/L	*pntAB↑*	Membrane bound proton translocating, pyridine nucleotide tranhydrogenase, reduces NADP +	AM1 medium with xylose	Biomass: < 0.1 g/L*; *pntAB* ~ 0.5 g/L	[71]
	15 mM	*fucO↑*	Reduces L-lactaldehyde to L-1,2-propanediol	Acid treated sugarcane bagasse	Ethanol titer: < 5 g/L*; *fucO* ~ 42 g/L	[105]
	1 g/L	*yghA↑*	Reductase activity toward butyraldehyde and decanal	AM1 with xylose	Ethanol productivity: ND*; *yghA* 0.72 g/L/h	[73]
	2 g/L	mutant *irrE↑*	Global regulator in radioresistance in *Deinococcus radiodurans*	Acid treated corn stover hydrolysate	Biomass: ND*; mutant *irrE* OD_600_ ~ 2.5	[74]
	10 mM	*ucpA↑*	Predicted acetoin dehydrogenase	AM1 medium with xylose	Ethanol titer: 0.55 g/L*; *ucpA* 40.3 g/L	[118]
	0.5 g/L	*cysC, cysH, cysM, cysN, cysQ, metA, metB, metC, sbp, tauA, tauB, tauC, tauD^*	*cysH, cysH, cysM, cysN, cysQ* – involved in sulfur metabolism; *metA, metB, metC* – de novo methionine biosynthesis; *sbp* – high affinity uptake of sulfate and thiosulfate; *tauA, tauB, tauC, tauD* – taurine uptake system	AM1 medium with xylose	Biomass: ND*; Cysteine supplementation ~ 1.5 g/L	[71]
*S. cerevisiae*	25 mM	*∆ZWF1, ∆GND1, ∆RPE1, ∆TKL1*	*ZWF1* – glucose-6-phosphate dehydrogenase; *GND1* – 6-phosphogluconate dehydrogenase; *RPE1* – D-ribulose-5-phosphate 3-epimerase; *TKL1* – transketolase	SD medium with glucose	WT parent ~ 20% growth inhibition*; Deletion of each gene resulted in at least 80% growth inhibition	[47]
	30 mM	*YAP1↑, GSH1↑, GLR1↑*	*YAP1* – basic leucine zipper (bZIP) transcription factor; *GSH1* – gamma glutamylcysteine synthetase, glutathione biosynthesis; *GLR1* – cytosolic and mitochondrial glutathione oxidoreductase	YPD medium with glucose	Biomass: OD_600_ < 0.1*; *YAP1* OD_600_ ~ 0.8; *GSH1* ~ 0.7; *GLR1* ~ 0.7	[54]
	2 g/L	*SPE3↑*	Spermidine synthase	Acid treated corn stover hydrolysate	Ethanol productivity: 0.13 g/L/h*; *SPE3* – 0.24 g/L/h	[85]
	4 g/L	*GLR1↑, OYE2↑, ZWF1↑, IDP1↑*	*OYE2* – FMN containing oxidoreductase; *IDP1* – mitochondrial isocitrate dehydrogenase	YPD medium with glucose	Biomass: OD_600_ ~ 0.8*; Overexpression of each gene separately ~ 1.0 Ethanol titer: ~ 35 g/L*; *IDP1, GLR1* ~ 45 g/L; *OYE1*, *ZWF1* ~ 40 g/L	[119]
	15 mM	*MSN2↑*	Stress-responsive transcriptional activator	SC medium with glucose	Biomass: OD_600_ ~ 3.5*; *MSN2* ~ 6.0	[120]
*Caldicellulosiruptor bescii*	15 mM	*bdhA↑*	Butanol dehydrogenase	LOD medium with maltose/cellobiose as carbon source	Biomass: 0.4 g/L*; *bdhA* 0.5 g/L	[95]
*Clostridium thermocellum*	10 mM	*bdhA↑, speE↑*	*speE* – spermidine synthase	CTFUD medium with cellobiose	Biomass: OD_600_ ~ 0.3*; co-expression of *bdhA speE* ~ 0.9 Ethanol titer: 6 mM; co-expression of *bdhA speE* 7 mM	[90]
*Candida tropicalis*	1 g/L	*ADH1↑*	Alcohol dehydrogenase	M9 medium with glucose	~ 60% furfural removed from medium by control*; 100% furfural removed from medium by overexpression of ADH1	[93]
*Zymomonas mobilis*	0.69 g/L	*udhA (co-expressed with ZMO1771)*	Soluble pyridine nucleotide transhydrogenase, reduces NAD^+^	Acid treated corn stover hydrolysate	Biomass: OD_600_ ~ 0.75*; co-expression of *udhA ZMO1771* ~ 1.20 Ethanol titer: ~ 21 g/L*; co-expression of *udhA ZMO1771* ~ 30 g/L	[91]
*Pseudomonas putida*	2 g/L (and 1 g/L 5-HMF)	*psJN↑*	Oxidoreductase, converts aldehydes into corresponding acid	Acid treated corn stover hydrolysate	Biomass: OD_600_ ~ 0.5*; *psJN* ~ 2.5	[103]
	20 mM	*PP_RS19785↑, PP_RS18130↑*	Putative ABC-type transporters with efflux activity	Hydrolysate conditions	Biomass: OD_600_ ~ 9-fold (*PP_RS19785*) and 3.6-fold (*PP_RS18130*) higher as compared to the control	[104]

^∏^Genes were expressed separately and case of co-expression is mentioned in the table. ND - signifies Not Detected. ^+^1 g/L 5-HMF was also present in the medium.

^*^Represents value of the control strain

^#^Percent increase in biomass as compared to the control strain

*↑*Indicates overexpression and ∆ indicates deletion

^Microarray results indicated downregulation of genes involved in sulfur assimilation in presence of furfural and the study tested supplementation with cysteine as a sulfur source in presence of furfural

Results of the studies in the earlier section suggest that the genes of the pentose phosphate pathway (PPP) could play a significant role in conferring tolerance against furfural. Analysis of gene disruption library of *S. cerevisiae* revealed that deletion of *ZWF1*, *GND1*, *RPE1* and *TKL1* result in an increase in doubling time as compared to the WT parent strain BY4741 [47]. In presence of 15 mM furfural the doubling time of WT strain was 4.3 (± 0.1) h while that of *ZWF1, GND1, RPE1* and *TKL1* mutants was 12.0 (± 0.9), 14.0 (± 4.0), 19.0 (± 2.0) and 13.0 (± 4.0) h, respectively. And in presence of 40 mM 5-HMF, the parent strain had doubling time of 7.5 (± 0.1) h while that of respective mutant was 19 (± 2.4), ∞, 29.0 (± 2.0) and 21.0 (± 2.1), respectively. Overexpression of the PPP committed gene *ZWF1* allowed the cells to grow at the lethal concentration of 50 mM furfural. Results highlight that increased carbon flow through the PPP results in improved tolerance against both furfural and 5-HMF. Overexpression of the *YAP1* transcription factor has also been involved in conferring tolerance against both furfural and 5-HMF [54]. At a concentration of 40 mM 5-HMF, the strain overexpressing *YAP1* was able to grow while the one overexpressing *ZWF1* was not able to increase its biomass. At 30 mM concentration of either furfural or 5-HMF, overexpression of *YAP1* was more beneficial in enhancing microbial growth as compared to overexpression of *ZWF1* gene in *S. cerevisiae*. In same study it was reported that an increase in the cellular glutathione levels by overexpression of *GSH1* and *GLR1* was also beneficial in increasing the tolerance against furfural. The results resonate with the earlier findings that furfural stress leads to an increase in oxidative stress by an increase in cellular ROS load [53]. And glutathione is the key player involved in maintaining cellular reducing environment where Gsh1 catalyzes the first step in *denovo* synthesis of glutathione via formation of L-ℽ-glutamylcysteine glycine from L-glutamate and L-cysteine. While Glr1 utilizes the reducing power of NADPH to regenerate reduced glutathione. Association of *YAP1* overexpression with more efficient tolerance could be explained by the fact that the increased *YAP1* transcripts levels have been observed under stress conditions such as heat shock [57], oxidative stress response [58, 59] and hydroperoxide stress [60]. Overexpression of *YAP1* can stimulate transcription of a diverse set of genes which can confer an efficient protective response from the cell in the face of multipronged furfural challenge (Fig. 2).

In *S. cerevisiae* strain NRRL Y12632 a novel aldehyde reductase gene was identified which exhibited NADPH dependent reduction of common lignocellulosic aldehyde inhibitors including furfural and 5-HMF. The partially purified protein Y63 exhibited specific activity of 4.19 (± 0.17) and 0.58 (± 0.07) U/mg protein against furfural and 5-HMF, respectively [61]. Interestingly, the importance of *ZWF1* gene in tolerance against hydrolysate derived phenolic compounds ferulic acid, 4-hydroxybenzoic acid and coniferyl aldehyde has also been reported [62]. Both ferulic acid (6 mM) and 4-hydroxybenzoic acid (25 mM) led to an increase in cytoplasmic ROS load while that from coniferyl aldehyde (1 mM) led to a localized ROS stress in mitochondria and to the endoplasmic reticulum. Overexpression of *ZWF1* gene resulted in a decrease in the ROS load. It further suggests that overexpression of a single gene *ZWF1* can be beneficial in conferring tolerance to both furan and phenolic class of inhibitors by increasing the cellular NADPH levels. It will be interesting to test co-expression of *ZWF1* with *YAP1* transcription factor and observe any resultant synergistic protective effect against furfural. To identify more causal genes responsible for tolerance against furfural and 5-HMF a genome analysis of strain NRRL Y12632 was performed [63]. Two genes—*ADH6* and *ADH7*—were identified to be important in conferring tolerance. Overexpression of *ADH6* and *ADH7* was important in rescuing growth in presence of 40 mM 5-HMF and not 40 mM furfural. The enzyme activities were determined for the purified proteins in presence of either NADH or NADPH. For furfural, Adh6 exhibited specific activity of 62.0 ± (2.0) mU/mg protein in presence of NADH and 97.7 ± (4.2) mU/mg protein in presence of NADPH; Adh7 exhibited specific activity of 86.1 ± (3.9) mU/mg protein in presence of NADH and no significant activity with NADPH as a cofactor. For 5-HMF, Adh6 did not exhibit any significant activity in presence of NADH as a cofactor and specific activity value of 78.7 ± (6.5) mU/mg protein was observed in presence of NADPH as a cofactor; Adh7 exhibited specific activity value of 157.4 ± (13.1) mU/mg protein in presence of NADH as a cofactor while no activity in presence of NADPH. It suggests that microbial response involves increasing the expression levels of dehydrogenases which can utilize either NADH or NADPH as cofactors to confer tolerance against the furan inhibitors. And the enzyme having lowest apparent *K*_*m*_ for the respective cofactor in presence of furfural plays a more important role in tolerance. The results are along expected lines since 5-HMF is relatively less toxic to microbial metabolism as compared to furfural.

For *E. coli* strains several studies have been pursued to identify the genes responsible for conferring tolerance against furfural. A genome wide study was conducted to identify genes conferring furfural tolerance in *E. coli* under a selection pressure of 0.75 g/L furfural [34]. About 6% of the total *E. coli* genes were highly expressed as compared to control and were categorized into processes associated with cell membrane, cell wall and biosynthesis. The clones harboring *thyA*, *lpcA* and *groESL* were effective in conferring tolerance against 0.75 and 1.50 g/L furfural. Function of *thyA* in conferring tolerance against furfural has been described in this text before [52]. *lpcA* catalyzes the first committed step in biosynthesis of lipopolysaccharide (LPS) core by using the PPP metabolite, D-sedoheptulose 7-phosphate, to form heptose which is incorporated in the inner core of LPS and likely contributes to repair the membrane damage caused by furfural. *groESL* encodes for a chaperone which helps in proper folding of proteins and is involved in conferring thermal stress tolerance and interestingly is also involved in conferring tolerance against butanol and ethanol [64] which makes it an important player in conferring tolerance across different stressors. Higher levels of GroESL will aid in proper refolding of the proteins as a repair mechanism to counter oxidative damage. In another study co-overexpression of *ahpC* and *yhjH* genes with *thyA* was also beneficial in conferring tolerance against furfural [33]. *ahpC* encodes for alkyl hydroperoxide reductase and is part of *ahpCF* operon. This operon is responsible for reducing peroxides to alcohols during ROS stress. These studies suggest that the microbial responses to offset the oxidative and thermal stresses are beneficial in conferring tolerance against the furfural and some of the targeted cellular processes are shared across these different stressors.

Another screening study was conducted to identify novel gene(s) involved in conferring tolerance against furfural. A plasmid based library screen consisting of multidrug efflux pumps, porins and select exporters from *E. coli* was undertaken [65]. Wherein small multidrug resistance pump SugE and MdtJI, and lactate/glycolate:H^+^ symporter LldP were involved in tolerance against furfural. Authors reported that overexpression of *lldP* was relatively toxic to cells while *sugE* provided minimal benefit. Overexpression of *mdtJI* with 0.01 mM IPTG, 1.25 g/L furfural and 10% xylose as carbon source produced around 40 g/L ethanol at 96 h, while the control strain with empty plasmid produced only around 20 g/L at the same time point. In addition, overexpression of *mdtJI* also increased cell viability by 3.5 and 1.6-fold during 48 and 72 h incubation, respectively, which indicates that an increase in microbial biomass is responsible for the higher ethanol titers under furfural treatment. In another study, the role of polyamines in conferring tolerance against furfural was also investigated [66]. Polyamines are organic compounds which can bind to DNA as well as cell membranes and serve as a protective function against stress. It was found that overexpression of *puuP* and *potE* was beneficial in conferring tolerance against 10 mM furfural in AM1 mineral media with 10% xylose as carbon source. Neither fermentation of xylose nor increase in biomass could be observed in *E. coli* strain LY180 harboring empty plasmid in presence of the inhibitor. When *puuP* and *potE* were overexpressed in presence of 10 mM furfural then fermentation was completed within 96 h with ethanol titers of around 43 g/L which were comparable with the control strain without any inhibitor.

However, excess levels of cellular NADPH pools have been reported to result in growth cessation in *E. coli* and increase in biomass resumes only when a NADPH intensive polyhydroxybutyrate (PHB) biosynthetic pathway is introduced in the cell [67]. This idea was successfully applied in order to confer furfural tolerance in ethanologenic *E. coli* strain SSK101. Authors knocked out *pgi* gene of the glycolytic pathway in order to force glucose flux via the PPP and furfural present in the medium served as a sink for excess NADPH. It was observed that the growth rate of SSK101 strain was proportional to the concentration of furfural in the medium until the sink for excess NADPH was balanced with the biosynthetic NADPH requirement of the cell. And any increase or decrease of furfural concentration from the optimum level led to a decrease in the specific growth rate. In presence of glucose-xylose mixture at a combined concentration of 5.5% and binary stress of 1 g/L each of furfural and 5-HMF, SSK101 produced ethanol at 77% of the maximum theoretical yield whereas no ethanol could be detected in the case of parent SSK42 strain under same cultivation conditions [68]. The observation can be explained due to an excess of cellular NADPH when furfural concentration is below the optimum level and a scarcity of NADPH when inhibitor levels were higher than the optimum concentration. In another study, *E. coli* LY180 was adapted to increasing concentration of furfural of up to 1.3 g/L in 54 serial transfers which resulted in strain EMFR9. Using microarray analysis and it was found that EMFR9 had ≥ twofold lower expression of *yqhD* and *dkgA* genes. Both purified proteins reduced furfural to furfuryl alcohol in the presence of NADPH as a cofactor and exhibited high affinity towards NADPH with apparent *K*_*m*_ value of YqhD being 8 µM and that of DkgA to be 23 µM. Overexpression of respective protein was detrimental to cell growth in presence of furfural and deletion of both was beneficial in increasing microbial biomass in presence of 1 g/L furfural [69]. It suggests that abundance of proteins utilizing NADPH as a cofactor for detoxifying furfural leads to a competition for NADPH between detoxification and biosynthetic processes where the process being catalyzed by the protein with lower apparent *Km* value gets preferential utilization of NADPH. The deletion of same genes—*yqhD* and *dkgA*—was also instrumental in increasing tolerance against 5-HMF [70]. In presence of 2.5 g/L 5-HMF, LY180 strain with deletion of *dkgA* and *yqhD* were able to increase in biomass as compared to parent LY180. Along similar lines, overexpression of *pntAB* was observed to be associated with tolerance against 2.5 g/L furfural in LY180 strain. *pntAB* is a membrane bound transhydrogenase which oxidizes NADH to reduce NADP^+^ and increases the cellular NADPH pools. This observation further adds to the existing knowledge of increasing NADPH pools for furan tolerance. In another study, to further elucidate the toxicity of furfural in LY180 strain, microarray analysis was conducted in presence of furfural. It was found that expression of genes involved in biosynthesis of purines, pyrimidines and amino acids was reduced by more than twofold [71]. Interestingly, expression of genes involved in cysteine and methionine biosynthesis increased under furfural stress. Expression of genes involved in sulfur assimilation—*cycC, cysH, cysI, cysM, cysN, cysQ, metA, metB, metC, sbp, tauA, tauB, tauC* and *tauD*—increased by more than twofold. To test the tolerance effect conferred by amino acid supplementation, 0.1 mM of each of the 20 amino acids were added to the cultures, separately and in presence of 1 g/L furfural. Only 5 amino acids were able to rescue growth at the tested concentration. In order of potency, they were cysteine > methionine > serine and arginine > histidine. It is important to mention that assimilation of sulfur is a NADPH expensive process and uses four moles of NADPH per mole of sulfur assimilated. In minimal (AM1) medium used in the study, sulfur is provided in sulfate form and needs to be reduced to hydrogen sulfide before it can be assimilated. Addition of amino acid reduces the biosynthetic requirement of the cell and subsidizes an increase in biomass in presence of the inhibitor.

Another strategy has been to deploy enzymes which utilize NADH to detoxify furfural and thus do not deplete the cellular NADPH levels. *fucO* is a native *E. coli* gene which is involved in fucose metabolism and encodes NADH dependent L-1,2-propanediol reductase. FucO catalyzes NADH dependent reduction of furfural and 5-HMF with apparent *K*_*m*_ values of 0.4 ± 0.2 mM and 0.7 ± 0.3 mM, respectively, with no activity in presence of NADPH as a cofactor [72]. In presence of 10% xylose, 15 mM furfural and 0.1 mM IPTG, overexpression of FucO reduced the lag phase as compared to the control strain with empty plasmid. Ethanol was produced at approximately 90% of the maximum theoretical yield with titers (> 40 g/L) which was similar to the strain carrying empty vector control in absence of furfural. Another example is overexpression of *ygha* gene which encodes for a putative oxidoreductase protein and preferentially utilizes NADH as a cofactor [73]. Ygha displayed an apparent *Km* value of 0.03 mM against furfural in presence of NADH and furfural. In presence of 10% xylose and 1 g/L furfural, *E. coli* strain SSK42 overexpressing YghA produced 5.3-fold higher ethanol with 97% efficiency as compared to the empty plasmid control under similar cultivation conditions.

An example of convergence stress tolerance against disparate stresses is exhibited by a transcriptional regulator *irrE* from radiation resistant *Deinococcus radiodurans*. IrrE is involved in RecA mediated DNA repair pathways in its native host which gives it a remarkable ability to repair double stranded DNA damage. Using error prone PCR, the *irrE* gene from its native host was amplified and transformed in *E. coli* [74]. After two step selection, strain F2-1 was selected for further studies. In presence of 2.32 g/L furfural, F2-1 displayed 16-fold increase in biomass as compared to control with empty plasmid. And at 1.74 g/L furfural it exhibited just half of the ROS load as compared to the control. The transformed strain also exhibited cross resistance against 5-HMF and vanillin. In presence of 4.35 g/L HMF, F2-1 exhibited 35-fold increase in biomass as compared to control while in presence of 1.5 g/L vanillin a 11-fold increase. In presence of 50% cellulosic hydrolysate, F2-1 exhibited a lag phase of only 8 h while the control had 36 h. When hydrolysate concentration was increased to 60%, F2-1 lag phase remained same while that of control stretched to at least 52 h. It is an indication, similar to *YAP1*, that transcription regulators exert a disproportionate influence on conferring tolerance to different aspects of stress encountered by a microbial strain under conditions relevant in industrial fermentations.

### Shared cellular targets between furfural and acetic acid

Acetic acid is a major organic acid inhibitor present in the hydrolysate which is formed due to deacetylation of the residues in the biomass during thermo-acidic treatment. Acetic acid has a relatively high *pK*_*a*_ of 4.8 (at 25 °C) which means that during fermentation conditions, where pH is commonly maintained between 6 and 7, a significant proportion of acid is in protonated form. Because of the lipophilic nature of the protonated form, it diffuses freely through the cell membrane and the circumneutral pH of the cytoplasm causes it to dissociate into CH_3_COO^−^ and H^+^. To prevent acidification of the cytoplasm the protons are pumped out of the cytoplasm via the plasma membrane ATPase which is at the expense of ATP generation due to loss of proton motive force. Effectively it results in rerouting of ATP from biosynthetic activities towards maintaining cellular homeostasis. It has been suggested that *S. cerevisiae* spends almost 10–15% of the cellular ATP pool towards maintaining the neutral pH as a result of cytoplasmic acidification caused by acetic acid [75]. In dilute acid hydrolysates of woody biomass consisting of deciduous trees concentration of acetate has been reported to be as high as 9 g/L while spruce hydrolysate harbors relatively lower concentration at 3 g/L. At any concentration of undissociated acetic acid greater than 5 g/L, the growth of *S. cerevisiae* is completely inhibited [40]. In terms of volume, acetic acid is the most abundant inhibitor as compared to furans. However, the higher concentration of acetic acid required to arrest microbial metabolism suggests that it is relatively a less potent inhibitor as compared to furfural.

### Glycolytic pathway

Like furfural, acetic acid also affects the activity of glycolytic enzymes in *S. cerevisiae*. The purified enzymes of glycolytic pathways were tested for inhibition by acetic acid and concentration required to cause 50% activity inhibition of the enzymes was determined [76]. It was reported that phosphoglyceromutase (122 mM) and enolase (120 mM) were among the most sensitive. The rest of the enzymes of the pathway with the 50% inhibition concentration are aldolase (172 mM), triosephopsphate isomerase (187 mM), glyceraldehyde-3-phosphate dehydrogenase (239 mM), phosphofructokinase (279 mM), phosphoglycerate kinase (357 mM) and pyruvate kinase (409 mM). The most resistant enzymes were hexokinase, glucose-6-phosphate, pyruvate decarboxylase and alcohol dehydrogenase which were all inhibited at concentrations > 1000 mM. To put the inhibition caused by acetic acid in a perspective with furfural, significant inhibition of glycolytic enzymes from *S. cerevisiae* was observed in presence of only 10 mM furfural [45]. In another study the influence of acetic acid on ATP metabolism in presence of glucose as sole carbon source, a respiratory deficient strain of *S. cerevisiae* was analyzed at pH 4.5. It was found that at 170 mM acetic acid concentration the yield of ATP was reduced by 30% as compared to the control in absence of acetic acid [77]. It is important to note that at the higher pH values of fermentation, which is commonly in the range of 6–7, the influence on cellular ATP levels would be more profound as a relatively higher proportion of species will be in the protonated form and diffuse across the cell membrane which will lead to increased loss of PMF.

### ABC-type transporters

ABC are ATP binding cassette type transporters which are commonly found in organisms and play a role in transport of molecules across the membranes by hydrolyzing ATP as an energy source. ABC-type transporters allow the microbial cell to transport molecules against their concentration gradient. Role of these transporters in antibiotic resistance is documented [78] and influence in conferring tolerance against lignocellulosic inhibitors also reported. Overexpression of ABC-type transporters in both *E. coli* and *S. cerevisiae* has been linked to tolerance against furfural and acetic acid, respectively. In presence of 60 mM acetic acid (pH = 4.0) and 20 g/L glucose, it was reported that deletion of the ABC transporter *PDR18* results in an extended lag phase of 40 h as compared to the 10 h lag phase in parental strain BY4741 [79]. The maximum transcription of *PDR18* occurs after exit of the cells from lag phase and transcript levels are threefold higher as compared to unstressed cells. Another study suggested deletion of a glycosylphosphatidylinositol-anchored cell wall protein Spi1p in *S. cerevisiae* being associated with sensitivity against different lipophilic acids [80]. Deletion of *SPI1* resulted in an increase of lag phase from 18 to 22 h in presence of 60 mM acetic acid. It was also reported that the *SPI1* mutant strain led to a decrease in cytosolic average pH from 6.9 to as low as 4.6 and overexpression of Spi1p conferred cell protection against different lipophilic acids ranging from (C_2_) acetic acid to (C_8_) octanoic acid. In case of *E. coli*, relatively low expression levels of a class of ATP transporters—SugE and MdtJI—led to increased tolerance against furfural [65]. The mentioned MDR transporters have been reported to have efflux activity against a range of lipophilic compounds [81] and this efflux activity seems to be responsible for transporting furfural outside cell.

### Influence on redox biology, oxidative stress and purine metabolism

The presence of acetic acid in the medium also leads to a redox imbalance in the cell and consequently decrease in NADPH required for an increase in biomass. At a concentration of 1 g/L, acetic acid results in reduction of NADPH/NADP^+^ ratio from 4.92 ± 0.2 to 3.54 ± 0.52 (p = 0.01) [82]. Modulation of oxidative stress response also leads to tolerance against acetic acid. Using a genomic library approach, *RCK1* gene was identified as beneficial in conferring tolerance against acetic acid [83]. In presence of 40 g/L xylose and 5 g/L acetic acid, *S. cerevisiae* strain SR8R overexpressing *RCK1* consumed 13 g/L of xylose within 96 h at the rate of 0.139 ± 0.001 g/L/h while no significant xylose metabolism could be detected in control strain SR8C. Strain SR8R also displayed significantly reduced ROS load as compared to the control strain SR8C under similar cultivation conditions. de novo purine biosynthesis has also been implicated in conferring tolerance against acetic acid [84]. *ADE1*, *ADE13* and *ADE17* genes are involved in de novo synthesis of purines. In presence of 100 g/L glucose and 5 g/L acetic acid, *S. cerevisiae* strains overexpressing Ade1, Ade13 and Ade17 recorded ethanol productivity of 1.21, 1.20, 1.55 g/L/h, respectively, as compared to 1.11 g/L/h of the control strain with empty plasmid. Remarkably, the ROS load of respective transformed strain also decreased by 21.04, 16.61 and 40.74%, in presence of the acetic acid. Weak acid stress also decreases the cellular ATP pools as mentioned earlier in this section. Overexpression of *ADE1*, *ADE13* and *ADE17* led to an increase of total adenyate pool consisting of ATP, ADP and AMP by 10.76, 18.91 and 33.29%, respectively. It is interesting to note that in the case of *E. coli* overexpression of purine biosynthesis gene confers tolerance against furfural. And furfural causes double stranded breaks in AT rich regions of plasmid DNA [49]. It appears that acetic acid affects DNA metabolism in *S. cerevisiae* with at least some shared characteristics with that observed in *E. coli*.

Overexpression of spermidine synthase (*SPE3*) has been tested in *S. cerevisiae* for tolerance against the furans and acetic acid inhibitors. In presence of 2 g/L each of furfural and 5-HMF the strain overexpressing *SPE3* had a lag phase of 72 h which was 33% shorter than that of the control strain (D452-2). While in presence of 3 and 4 g/L acetic acid the strain displayed 96 and 16% higher maximum specific growth rate, respectively, as compared to the non-transformed parent strain. The ethanol productivity of the transformed strain further increased upon disruption of the polyamine transport protein (Tpo1) in the extracellular medium which results in an increase in cellular spermidine levels. The resultant strain exhibited 85% higher ethanol productivity as compared to the parent strain in presence of 2 g/L each of furfural and 5-HMF [85].

### Furfural tolerance studies in other microbial strains

As described in this section, the metabolic strategies utilized for conferring tolerance against furfural in *E. coli* and *S. cerevisiae* have also been successful in engineering tolerance in other microbial catalysts with relevance in bioenergy studies which includes cellulolytic organisms. It suggests that at the molecular level the microbial response against furfural across different classes of biocatalysts is conserved.

Cellulolytic microbes have an important role to play in bioenergy studies with their intrinsic ability for simultaneous saccharification and fermentation (SSF) [86]. Members of *Clostridium* spp. have been successfully utilized in bioproduction of metabolites of interest [87, 88]. It is interesting to note that furfural tolerance studies have been performed in cellulolytic microbes as members of this group harbor the ability to solubilize the sugars efficiently (at least theoretically) from lignocellulosic biomass without the need for pretreatment. Along the lines of manipulation of redox balance in *E. coli* and *S. cerevisiae*, overexpression of a heat stable NADPH dependent butanol dehydrogenase gene (*bdhA*) from *Thermoanaerobacter pseudethanolicus* 39E in *Clostridium* spp. was instrumental in imparting furfural tolerance. Overexpression of the *bdhA* gene in *Clostridium thermocellum* 1313 in presence of 10 mM of either furfural or 5-HMF results in 30–35% and 54–84% improved growth, respectively, as compared to the control strain [89]. Overexpression of *bdhA* gene in *C. thermocellum* has also been demonstrated to confer tolerance against acetic acid. In presence of 15 mM acetic acid, the *bdhA* overexpressing strain exhibited both higher cell density and ethanol titer by 34 and 43%, respectively. In the same study, the synergistic effect of co-expression of *bdhA* and *speE* on thermotolerance of *C. thermocellum* JWCT16 was also observed which showed 69% higher optical density as compared to the control strain [90]. A significant interpretation of these three studies is that increased requirement of NADPH for furfural detoxification does not lead to a detrimental effect on increase of biomass of cellulolytic microbes. It is possible that as compared to facultative aerobes, strictly anaerobic cellular metabolism is better suited to compensate for an increased NADPH requirement. In *Zymomonas mobilis* ZM4 overexpression of NADPH dependent ZMO1771 and the soluble transhydrogenase *udhA* has also been demonstrated to be involved in conversion of furfural and 5-HMF into their respective less toxic alcohols [91].

The spermidine biosynthesis pathway when engineered in *C. thermocellum* also results in higher tolerance to furans and acetic acid and increased ethanol titers. In a strain deficient in spermidine biosynthesis *(ΔspeE*), addition of 1 mM spermidine led to an increase in biomass and ethanol by 16 and 19%, respectively, as compared to the non-supplemented control in presence of 10 mM furfural. Under same amount of spermidine and in presence of 10 mM 5-HMF, the increase in biomass was 19% while 35% higher ethanol titers were observed when supplemented with 2 mM spermidine. The tolerance was even more profound (40% more biomass) when 1 mM spermidine was added to medium containing 10 mM acetic acid and 23% higher ethanol titers were observed as compared to the control [92]. In the case of *Candida tropicalis* when treated with 3, 5 and 7 g/L furfural concentrations the transcription levels of *adh1* were observed to be higher as compared to the untreated control. The transcript levels decreased upon complete removal of furfural from the medium which suggests that either the dehydrogenase is directly involved in detoxification of furfural or the cell responds to altered redox requirements by modulating the expression of the gene. In the same study the authors showed that the NADH/NAD^+^ ratio was lowered in the strain treated with 3, 5 and 7 g/L concentrations of furfural [93]. Similar effect of furfural on lowering cellular NADH/NAD^+^ ratio in *S. cerevisiae* has also been described earlier [55]. In a transcriptomic response study of *C. thermocellum* under multimeric stress of furfural, heat and ethanol it was reported that the highest down regulation of genes under furfural treatment was observed in the group corresponding to sulfate transporter subunits and enzymes involved in sulfur metabolism. These group of genes were also repressed in *E. coli* under furfural stress [71] and downregulated to a lesser extend in the heat and ethanol stress [94]. In an earlier study [95], overexpression of NADPH dependent dehydrogenase in *C. thermocellum* did not lead to a decrease in biomass accumulation suggests that the scarcity of NADPH is most likely not the reason for reduced expression of sulfur assimilation genes in *C. thermocellum*. It is possible that furfural inhibits expression of genes involved in sulfur assimilation by interaction with an activator involved in transcription of the genes in the cellulolytic microbe.

Overexpression of *bdhA* in *Caldicellulosiruptor bescii* allowed it to metabolize 15 mM of furfural and 5-HMF with simultaneous increase in biomass at 75 °C [95]. It is an interesting observation as mesophilic microbes have been reported to first metabolize the respective furan aldehyde into the corresponding furan alcohol before any increase in microbial biomass could be observed. A novel fungal species *Amorphotheca resinae* ZN1 has also been reported to sequentially detoxify furfural, 5-HMF and acetic acid in a growth medium consisting of dilute sulfuric acid treated corn stover. Interestingly, the novel microbe can utilize furfural for both growth and respiration. Under aerobic conditions strain ZN1 was able to completely detoxify 1 g/L furfural within 64 h. While under anaerobic conditions only 35% of furfural was detoxified by the said time point [96]. In the detoxified hydrolysate the strain utilized 55 g/L glucose to produce around 40 g/L ethanol while the non-detoxified medium < 10 g/L ethanol was produced within 60 h [97]. The potential of this novel fungal strain to be used as chassis for bioproduction of diverse industrial compounds needs to be explored further.

*Pseudomonas putida* has also emerged as a promising workhorse with wide potential in industrial biotechnology [98, 99]. It’s metabolic pathway has been characterized [100] and the microbe has potential to bioproduce polyhydroxyalkanoates, polyketides, rhamnolipids, terpenoids [100]–[102]. Heterologous expression of furan metabolizing PsJN enzymes from *Burkholderia phytofirmans* in the industrially important strain *P. putida* KT2440 strain has been reported to result in utilization of both furfural and 5-HMF as a carbon source to both grow and respire. In a culture medium consisting of 50% acid -treated corn stover hydrolysate, the *psJN* expressing strain, at 36 h, displayed OD_600_ value as ~ 2.5 while the control value was ~ 0.5 which represents a fivefold increase in the biomass [103]. In order to further identify the causal genes for furan tolerance in *P. putida*, strain KT2440 was adapted in M9 minimal medium with 1 g/L furfural concentration and led to identification of putative genes with homology to ABC-type transporters. KT2440 strains overexpressing either PP_RS19785 or PP_RS18130 genes with resemblance to ABC-type transporters led to a 9 and 3.6-fold increase in biomass in a medium resembling hydrolysate with furfural concentration at 20 mM [104]. These results make *P. putida* a promising candidate for conversion of lignocellulosic carbon into compounds of industrial utility.

### Future directions

In order to achieve carbon neutral as well as cost efficient carbon conversion into an industrial compound of interest the microbial strain should be able to withstand the stressful conditions present in an industrial fermenter with lignocellulosic hydrolysate as a carbon source. The mechanism of furfural toxicity and strategies to engineer tolerance is conserved across prokaryotes (*E. coli*) and eukaryotes (*S. cerevisiae*). In Bacteria Domain the strategies to engineer tolerance are again conserved across different phyla as evidenced by members of Pseudomonadota (*Escherichia* genera) and Bacillota (*Clostridium* genera). In both *E. coli* and *S. cerevisiae*, the fermentability of lignocellulosic hydrolysate has been positively correlated with tolerance to furfural [40, 105]. Furfural targets a variety of cellular functions which are important in maintaining homeostasis and these targets are shared with other stresses and toxins. Thus, a furfural tolerant microbial strain should be able to efficiently repair the extensive cellular damage which can also be caused by other toxins present in the hydrolysate. Importantly, the robust strain should harbor an efficient lignocellulose carbon to pyruvate conversion pathway in presence of inhibitors. The pyruvate can be channeled to any metabolite of interest upon appropriate genetic intervention. More widespread application of furfural tolerance engineering in existing as well as new emergent industrial strains harbors a potential way to tap into the cheaply available biomass carbon as a cost-efficient source of carbon for microbial metabolism on a large industrial scale. A few strategies are described below.

### Balanced NADPH production

In preceding sections, the importance of excess cellular NADPH levels to overcome furan stress is highlighted in multiple independent studies [47, 68, 71]. To harvest the excess reducing power of NADPH for industrial biosynthesis of compound of interest, without compromising on the biomass increase, a few coupled gene expression approaches could be investigated. *Firstly*, a direction is shown by the study [68] where the *pgi* gene was deleted in order to direct the glucose flux towards the pentose phosphate pathway. The excess NADPH was instrumental in reducing the lag growth phase in presence of the furan inhibitors. In presence of 0.6% glucose the strain had higher biomass productivity and achieved its highest biomass at 120 h in the presence of furan inhibitors. Interestingly, with an increase in glucose concentration to 1.2% the biomass productivity was reduced, and the maximum value was obtained at 168 h. Biomass productivity at relatively higher glucose concentration was reduced due to exhaustion of the sink (furan inhibitors) from the medium. The growth dynamics in such a scenario can be improved by introducing an additional sink for the NADPH at a time which coincides with exhaustion of the inhibitor sink. In practice it can be achieved by inducing an industrially favorable and NADPH intensive biosynthetic pathway like for hydrocarbon synthesis. *Secondly*, oxidative stress is a common stress encountered by the microbial cell under normal metabolic functions and is exacerbated by inhibitors and high osmotic pressure exerted by sugar [106] and solid loadings [107]. Increased expression of NADPH dependent proteins involved in regeneration of reduced glutathione and thioredoxin under oxidative stress can be coupled with the excess cellular NADPH levels. The advantage of such a ‘generalist’ approach will be towards generation of a microbial strain which shall be robust against the common ROS stress encountered by any microbial strain under common industrial fermentation conditions. Such a microbe can be used as a base strain to engineer metabolic pathway of interest while utilizing the hydrolysate as a growth medium. The success of these strategies will lie in precisely engineering the balance between the source and sink and any deviation from the balance shall result in either a scarcity or a glut of the NADPH which in turn will be detrimental to the increase in biomass.

### Adaptive laboratory evolution (ALE)

ALE has emerged as a relatively quick method to develop desired characteristics in microbes of interest. This generalized strategy involves passaging the strain of interest in presence of unfavorable conditions in the growth medium. The unfavorable condition can be the presence of a toxin/inhibitor in the medium at sub lethal amount whose concentration is increased in a gradual stepwise manner during the evolutionary period. Commonly the genomes of the evolved strain are sequenced and in order to investigate the cause and effect-based hypothesis the mutations of interest are reverse engineered into the unevolved parent strain and then evaluated for appearance of the desired characteristic(s). This strategy has resulted in improved sugar utilization [108, 109], engineering novel metabolic pathways [110], release of carbon catabolite repression exerted by glucose on xylose utilization [111, 112], cross resistance to different stress conditions [113], resistance to lignocellulosic hydrolysate [114] and tolerance to octanoic acid [115]. These are only a few examples to highlight the widespread utility of this evolutionary technique.

The growth deficient excess NADPH producing strain can also be passaged in presence of a chemical or a NADPH intensive biosynthetic pathway. In this manner, the cellular machinery of the strain would evolve to balance the excess cellular NADPH load. If a chemical like furfural, which affects a variety of cellular functions, is selected to serve as an ‘evolutionary sink’ then it can also lead to a phenotype more efficient in repairing the damage caused to the diverse metabolic targets of furfural. This trait has potential to develop cross resistance against different stressors with shared cellular targets and relevance in industrial fermentation processes. In theory, a microbe evolved in such a manner should be robust and able to grow efficiently only in presence of a sink. An estimation of the carbon flux through the central metabolic pathways under the starting evolutionary conditions shall be helpful in designing an evolutionary approach.

### Use of cellulolytic anaerobes

The role of cellulolytic anaerobes can also be further tested in thermo-acidic treated biomass. Representative thermophilic cellulolytic anaerobe *Clostridium* spp. can serve as a promising candidate for co-culture fermentation strategy. The relatively dilute acid treatment results in dissolution of the pentose arabinoxylan fraction into the soluble part while the glucan fraction remains in the insoluble part and needs to be enzymatically treated in order to solubilize the hexose sugars. Using this methodology, the total glucose and total xylose recovery from acid treated rice straw has been reported to be 89.4 and 56.9%, respectively. And the total sugar recovery was 77.7% [17]. However, this enzymatic treatment step increases the processing cost due to use of purified enzyme cocktail consisting of β-glucosidase, endo-cellulase and endo-xylanase activities. In a study it has been observed that at the culture of *Clostridium thermocellum* can solubilize both glucan and xylan fractions of switchgrass at 65 ± 3 and 64 ± 6% efficiency, respectively [116]. This observation suggests that the SSF capability of inhibitor tolerant *Clostridium* can be utilized for efficient conversion of the hexose fraction of the lignocellulosic hydrolysate without any enzymatic treatment. *Clostridium* spp. lack a native metabolic pathway for utilization of pentose sugars [117]. Thus, the pentose fraction can be efficiently converted by either a *S. cerevisiae* or *E. coli* strain of interest. For efficient utilization of the pentose fraction it will be important to utilize a strain which is relieved from the carbon catabolite repression in order to prevent the diauxie in sugar utilization during the fermentation process.

## Conclusions

Achieving a carbon neutral bioproduction of compounds with industrial relevance is an important requirement of sustainable development where abundantly available lignocellulosic biomass has a more important role to play than presently realized. This review highlights the importance of developing microbial strains which can maintain optimum metabolic activity in the presence of multiple stresses during fermentation conditions. Furfural has the potential to be used as a model stressor in order to engineer tolerance against the multiple cellular stresses encountered by microbial strains under industrial fermentation conditions. The stress tolerant strains will harbor a glycolytic pathway which can efficiently convert lignocellulosic carbon into pyruvate under stressful conditions. The pyruvate can in turn be channeled to desired metabolite of interest upon required genetic manipulation in the microbial strain. Large scale utilization of robust microbial strains to synthesize high value industrial compounds will help to bring down the production costs of same by use of relatively cheap biomass as carbon source.

## Data Availability

All data is referenced herein.

## Abbreviations

5-HMF: 5-Hydroxymethyl furfural
G3P-DH: Glyceraldehyde-3-phosphate dehydrogenase
DH: Dehydrogenase
AlDH: Aldehyde dehydrogenase
PDH: Pyruvate dehydrogenase
NADH: 1,4-Dihydronicotinamide adenine dinucleotide
NAD +: Nicotinamide adenine dinucleotide
PPP: Pentose phosphate pathway
DNA: Deoxyribose nucleic acid
dUMP: 2'-Deoxyuridine-5'-monophosphate
dTMP: Thymidine-5’-phosphate
NADPH: Dihydronicotinamide adenine dinucleotide phosphate
ROS: Reactive oxygen species
DW: Dry weight
WT: Wild type
LPS: Lipopolysaccharide
IPTG: Isopropyl β-D-1-thiogalactopyranoside
ATP: Adenosine triphosphate
ALE: Adaptive laboratory evolution
ABC-type: ATP binding cassette-type
PHB: Polyhydroxy butyrate
